# Recent Advances in Pepper Fruit Glossiness

**DOI:** 10.3390/genes16111319

**Published:** 2025-11-02

**Authors:** Zongjun Li, Hu Zhao, Zihuan Jing, Zengjing Zhao, Meng Wang, Mingxia Gong, Xing Wu, Zhi He, Jianjie Liao, Mengjiao Liu, Zhiyang Ling, Risheng Wang

**Affiliations:** 1 Institute of Vegetable Research, Guangxi Academy of Agricultural Sciences, Nanning 530007, China; lizongjun.gxaas@gmail.com (Z.L.); zh107100421@126.com (H.Z.); nkyzzjing@163.com (Z.Z.); wmm43616@126.com (M.W.); ff9903@126.com (M.G.); wxscs@gxaas.net (X.W.); rf0073244455@163.com (Z.H.); 2 School of Agricultural Engineering, Guangxi Vocational University of Agriculture, Nanning 530009, China; jingzh@gxnzd.edu.cn (Z.J.); liumj2025@126.com (M.L.); 3 College of Agriculture and Biology, Guangxi Minzu Normal University, Chongzuo 532200, China; liaojianjie@gxnun.edu.cn; 4 School of Agricultural Engineering, Guangxi Vocational & Technical College, Nanning 530226, China; lingzhiyang.123@163.com

**Keywords:** pepper fruit, glossiness, epicuticular wax biosynthesis, cuticle, carotenoids

## Abstract

Pepper (*Capsicum frutescens* L.) is a globally important vegetable crop whose fruit glossiness serves as a key quality trait influencing consumer preference and market value. This review summarizes the measurement methods, influencing factors, and molecular regulatory mechanisms of pepper fruit surface glossiness, as well as the correlation between post-harvest changes in carotenoid content and fruit surface glossiness, aiming to provide references for the molecular breeding of high-gloss pepper cultivars. Pepper fruit glossiness is primarily determined by cuticle structure and composition. The content and arrangement of cuticular crystals significantly affect the specular reflection and diffuse reflection on the fruit surface. The ordered arrangement of long-chain alkanes enhances the anisotropy of specular highlights, reduces the contrast of diffuse reflection, and forms a high-gloss surface. In contrast, the imbalance of wax components or disordered accumulation of crystals leads to increased light scattering, resulting in a matte phenotype. Furthermore, carotenoid content strongly correlates with L*, a*, and b*, critically influencing fruit color intensity and hue. Currently, there are still several issues in the research on pepper glossiness, including the lack of standardized measurement methods, unclear gene regulatory networks, and unknown pathways related to post-harvest gloss maintenance and environmental responses. In the future, we should promote the combination of multiple technologies to establish unified measurement standards; integrate multi-omics to identify key genes; develop targeted preservation technologies based on the law of fruit gloss degradation; and breed pepper cultivars with high glossiness and good storage performance.

## 1. Introduction

Pepper (*Capsicum frutescens* L.), a vegetable crop within the Solanaceae family, is a globally significant economic crop. Over 40 pepper species were recognized as of 2022, though most remain wild or semi-wild. The genus comprises five principal cultivated species: *Capsicum annuum* L., *C. frutescens* L., *Capsicum chinense* Jacq., *Capsicum baccatum* L., *Capsicum pubescens* Ruiz & Pav. Among these, the first three species are cultivated worldwide, while the latter two are primarily grown in Central and South America [1,2]. The most commonly cultivated species in production is *Capsicum annuum* L. In recent years, pepper cultivation in China has exceeded 2 million hectares, representing 8–10% of the total vegetable planting area. Its output value and economic benefits rank highest among vegetable crops in China [3].

Fruit appearance, encompassing shape standardization, uniform coloration, and skin glossiness, constitutes the primary visual quality trait in peppers. Varieties with standard shapes, glossy skins, and vibrant colors, such as bell peppers and fruit-type peppers, are highly favored by consumers and offer significant economic benefits [4]. Bright red peppers command higher prices than dark red, orange, or yellow varieties, dark red fruits retain their color longer during storage compared to lighter-colored varieties [5]. Critically, pepper fruit gloss profoundly influences perceived freshness, ripeness, and texture, thereby determining overall quality perception and consumer preference through both sensory and psychological pathways, which ultimately governs fruit marketability.

Recent research has elucidated the fundamental mechanisms governing fruit gloss formation. Advanced imaging techniques, including confocal microscopy and scanning electron microscopy, have delineated the critical roles of epidermal wax crystals and cuticular wrinkles in determining fruit gloss appearance. Molecular investigations have identified key genes and enzymes responsible for wax and cutin biosynthesis and deposition, thereby clarifying the genetic basis of gloss development in diverse fruit species including cucumber, tomato, eggplant, and citrus [6,7,8,9,10,11]. Multiple studies demonstrate that dull fruit epidermis in cucumber constitutes a dominant trait governed by a single locus, whereas the glossy phenotype exhibits recessive inheritance [12,13]. The gene controlling fruit glossiness modulates epicuticular wax and cutin deposition on the extracellular surface of epidermal cell walls [14].

Compared to model crops like tomato and cucumber, research on pepper fruit glossiness remains substantially understudied. Key regulatory genes and pathways mediating environmental responses are largely unidentified. This review summarizes the research progress on the influencing factors, measurement methods, molecular regulatory mechanisms, and post-harvest glossiness of pepper fruit glossiness. It aims to further enhance the market economic value of pepper varieties with high-gloss phenotypes, and provide references for research on the freshness of peppers during transportation and storage, as well as subsequent breeding applications.

## 2. Pepper Fruit Surface Glossiness

### 2.1. Definition of Glossiness

Glossiness denotes the capacity of fruit surfaces to reflect incident light, with higher reflectance corresponding to increased gloss intensity. This optical property influences not only visual appearance but also correlates with fruit quality attributes including flavor and nutritional composition [15,16]. High gloss pepper fruit exhibit intense coloration and serve as visual indicators of freshness, adequate moisture content, and optimal nutritional status. Conversely, low gloss pepper fruit frequently develop a dull appearance with surface shriveling, typically resulting from physiological senescence, desiccation, or incipient spoilage.

**Figure 1:**
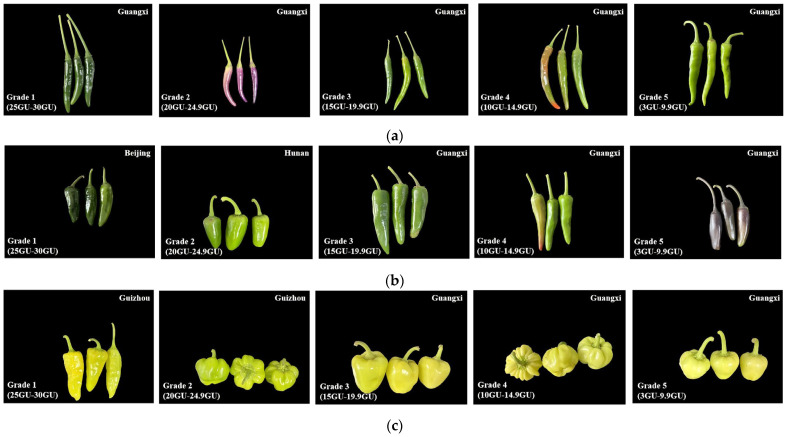
Schematic diagram of glossiness classification for different varieties of pepper in different regions of China: (**a**) *Capsicum annuum* L. var. *longum* Sendt. (sheep born shaped); (**b**) *Capsicum annuum* L. var. *longum* Sendt. (cattle born shaped); (**c**) *Capsicum annuum* L. var. *grossum* (L.) Sendt.

Recently, the Guangxi Academy of Agricultural Sciences has proposed a group standard for the determination method of pepper surface glossiness (Figure 1). The standard specifies that glossiness is defined as the quantified value of the specular reflection ability of the fruit surface to incident light, which is measured by a standard glossmeter at a 60° incident angle, and the results are expressed in Gloss Unit (GU) [17]. A GU range of 25.0–30.0 corresponds to Grade 1 (high gloss) for pepper fruit glossiness; a GU range of 20.0–24.9 is designated as Grade 2 (medium-high gloss); a GU range of 15.0–19.9 is classified as Grade 3 (medium gloss); a GU range of 10.0–14.9 refers to Grade 4 (medium-low gloss); and a GU range of 3.0–9.9 is defined as Grade 5 (low gloss).

### 2.2. Methods for Measuring Glossiness

Current assessment of early-stage fruit glossiness primarily relies on sensory analysis [18]. While this approach enables qualitative evaluation through experiential judgment, measurement repeatability suffers substantially from variable ambient lighting, viewing angle inconsistencies, and inter-observer variability.

**Figure 2:**
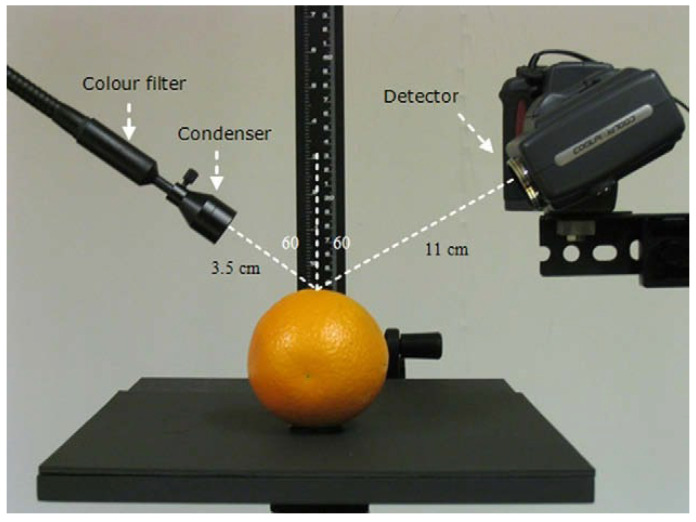
Design of gloss imaging system [18].

**Figure 3:**
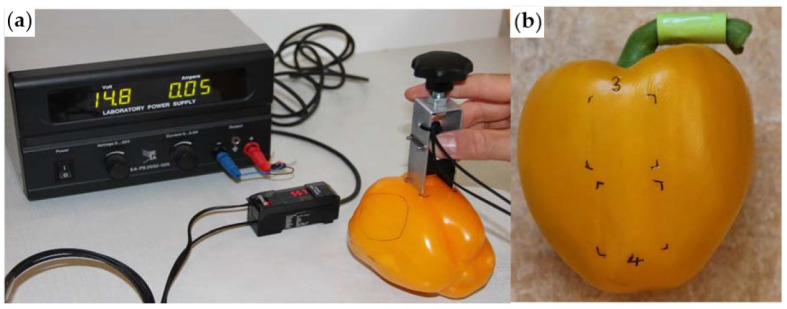
(**a**) Gloss measurements using designed machined holder; (**b**) marked spots/areas for repeated non-invasive measurements at the same positions [19].

**Figure 4:**
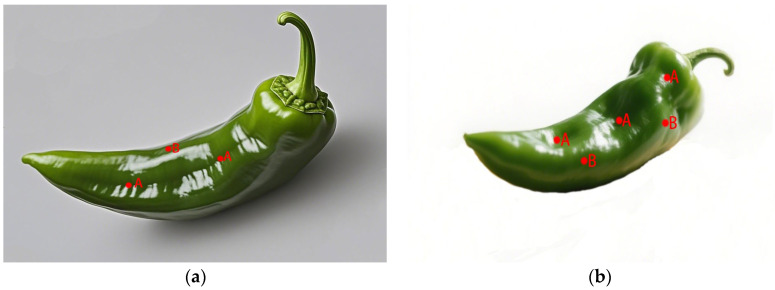
(**a**) Schematic diagram of test position locations for shallow rib groove-type pepper; (**b**) schematic diagram of test position locations for deep rib groove/wrinkled-type pepper. Note: Red dots indicate the locations of the test positions; Point A is located at the edge of the rib groove, and Point B is located at the ridge line.

In recent years, standardized gloss measurement instruments have been developed for agricultural products. Mendoza et al. developed a Gloss Imaging System (Figure 2) specifically for quantitative surface gloss analysis of fruits and vegetables [19]. Althaus and Blanke [20] designed a custom apparatus integrating a colorimeter, spectrometer, and VR-5200 profilometer to measure pepper fruit glossiness, with simultaneous acquisition of RGB image outputs (Figure 3). The Guangxi Academy of Agricultural Sciences performed multi-point measurements on pepper fruit samples using a gloss meter. The fruit surface types included smooth type, shallow-ridged type, and deep-ridged/wrinkled type (Figure 4). The surface glossiness was calculated via a data correction model, followed by repeatability verification and outlier data determination to obtain the GU values.

Furthermore, characterization of pepper fruit color has transitioned from qualitative sensory descriptions toward quantitative colorimetric analysis. Kasampalis et al. employed digital imaging to monitor bell pepper surface color evolution through CIE-Lab parameters for maturity assessment [21]. Wang et al. quantified color attributes using colorimetry, establishing that higher L* correspond to increased surface brightness [22]. Non-contact image recognition systems integrated with artificial intelligence vision technologies demonstrate significant potential. Convolutional neural network-based deep learning achieved 86.67% accuracy in simultaneous color and morphology identification of chili peppers [23]. Table 1 summarizes various testing methods, along with their advantages, disadvantages, and application scenarios.

In summary, current research emphasizes fruit color parameters, while standardized methodologies for surface gloss measurement remain underdeveloped. Future efforts should prioritize overcoming limitations in non-destructive detection technologies, developing specific gloss instrumentation for pepper fruit, and establishing evaluation standards based on optical properties or image analysis to advance sensory quality breeding programs.

### 2.3. Factors Affecting Glossiness

#### 2.3.1. Surface Appendages

Fruit surface appendages primarily comprise bloom [14], epicuticular wax [24] and trichomes [25]. Fruit bloom, a white powdery secretion, consists of silicate compounds synthesized by epidermal trichomes. Epicuticular wax and bloom destroy the smoothness of the fruit surface, generating a rough surface [26,27]. Incident light undergoes enhanced scattering and refraction across this irregular surface, diminishing specular reflection and consequently reducing gloss intensity [28]. Pepper trichomes development is governed by an incompletely dominant locus on chromosome 10 [29]. Among the five cultivated species, only *C. pubescens* Ruiz & Pav. exhibits epidermal trichomes, representing a unique germplasm resource. These trichomes increase surface contact points for atmospheric particulate adhesion, generating roughness and ultimately diminishing surface glossiness.

Trichome presence is a qualitative trait governed by a single nuclear gene that is inherited in a dominant-to-recessive ratio of approximately 3:1 [30]. This conclusion is highly similar to the inheritance pattern of the fuzz gene in cucumber [31]. Through bacterial artificial chromosome library construction and map-based cloning, Kim et al. Localized the key gene locus *Ptl1* on chromosome 10 that controls trichome formation [32]. Further studies have shown that the gibberellin receptor gene *GID1B* up-regulates *MYB* and *AP2/ERF* transcription factors, thereby orchestrating epidermal cell differentiation into trichome structures [29].

#### 2.3.2. Wax and Cuticle

**Figure 5:**
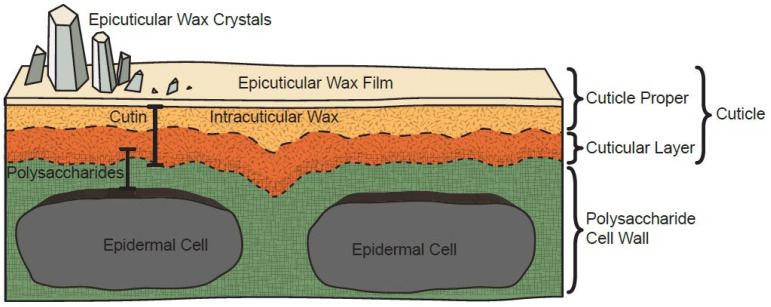
Plant cuticle structure [34].

The cuticle (Figure 5) is a unique hydrophobic lipid layer covering the outer surface of plant epidermal cells, composed of cutin and wax [33,34]. Wax is divided into intraculticular wax embedded within the cuticle matrix and epiculticular wax deposited as crystalline or amorphous films on the fruit surface. It is mainly composed of very-long-chain fatty acids (VLCFAs) and their derivatives (hydrocarbons, aldehydes, alcohols, esters, etc.), and also contains a small amount of non-VLCFA derivatives, such as terpenoids, flavonoids, sterols, etc. Wax confers diverse surface properties to fruits and is responsible for glossy appearance in many fruit species [35].

The cuticular layer, situated externally to the polysaccharide cell wall of epidermal cells, serves as the primary deposition site for wax. Cutin, a cross-linked polyester polymer derived from hydroxy and epoxy fatty acids, forms the structural framework of cuticle layer [36]. It is mainly responsible for viscoelastic properties characterized by low elastic modulus and high extensibility, collectively determining its mechanical rigidity [37].

Epicuticular wax crystals form amorphous films or diverse crystalline microstructures (e.g., flakes, rods, tubes, filaments, blocks), encompassing up to 23 distinct morphological types [38]. These structures range in size from submicrometer to micrometer scales [39]. These microstructures influence light reflection from the cuticle surface, directly determining fruit surface glossiness.

Pepper epicuticular wax consists primarily of long-chain n-alkanes, VLCFAs, silicic acid compounds, triterpenes, and minor sterols and flavonoids [40,41]. Long-chain n-alkanes, such as C31, typically assemble into ordered crystalline structures [42]. Vertically aligned epicuticular wax crystals can reduce light scattering, thereby enhancing surface glossiness. A moderate wax layer thickness can optimize the balance between light transmission and reflection. Excessive thickness induces turbidity through internal light scattering, while insufficient thickness fails to form an effective reflective layer [43]. The smooth genotype PI 257145 exhibited 6 times higher cutin content than its matte counterpart PI 224448, with 12 different cutin monomers accumulating at higher levels in PI 257145 [44].

In summary, wax content may not be the key factor directly affecting glossiness, rather, the composition, proportion, and crystal structure of wax exert greater influence on epidermal glossiness. The cuticle content governs the contrast and anisotropy of diffuse reflectance, thereby significantly contributing to the visual perception of fruit glossiness.

#### 2.3.3. Fruit Pigment Accumulation

Pepper fruit coloration is primarily governed by the composition and concentration of chlorophyll, anthocyanins, and carotenoids [45]. In fresh pepper fruit, quantification methods include pigment analysis via liquid chromatography and direct colorimetric measurement of fruit color value index (L*, a*, b*) [46,47]. In the pepper fruit color mutant E55, carotenoid accumulation peaked during color transition and ripening, coinciding with complete chlorophyll absence. This pigment profile correlated with maximal L values [48].

The red coloration of processed peppers is primarily attributed to capsanthin accumulation. Increased capsanthin content in ripened fruit enhances both color vibrancy and processing quality metrics [49]. Furthermore, capsanthin content determines red color intensity in mature pepper fruit [50]. He et al. [51] quantified peel color in 26 pepper cultivars using CIELAB color space parameters, demonstrating significant positive correlations between red color intensity and carotenoid accumulation.

Pigments in pepper fruits undergo deposition within cytoplasm. Their crystallization state and accumulation intensity directly influence epidermal light absorption and reflection efficiency. Enhanced pigment accumulation typically attenuates surface glossiness, attributed to reduced light-scattering capacity. Notably, carotenoid accumulation intensity exhibits an inverse correlation with L* [52,53], with high-carotenoid red and orange cultivars frequently displaying matte epidermal textures.

## 3. Molecular Regulatory Mechanism of Pepper Fruit Glossiness

Existing studies on fruit glossiness primarily focus on model species like cucumber [14] and tomato [54]. Due to the absence of systematic investigations in pepper fruit, core regulatory genes for gloss phenotypes remain undefined. Consequently, underlying genetic mechanisms must currently be inferred through indirect morphological proxies, including epidermal trichomes, wax deposition, cutin composition, and pigment accumulation patterns.

### 3.1. Biosynthesis and Transport of Wax and Cutin

Alterations in the wax biosynthesis pathway, due either to mutations in genes encoding wax synthases or to changes in their expression levels, can affect fruit glossiness. Numerous enzymes involved in the synthesis of cuticular components have been identified and characterized in the model plant Arabidopsis thaliana. To date, more than 190 genes have been implicated in the biosynthesis and transport of cuticular wax in Arabidopsis [55,56,57].

**Figure 6:**
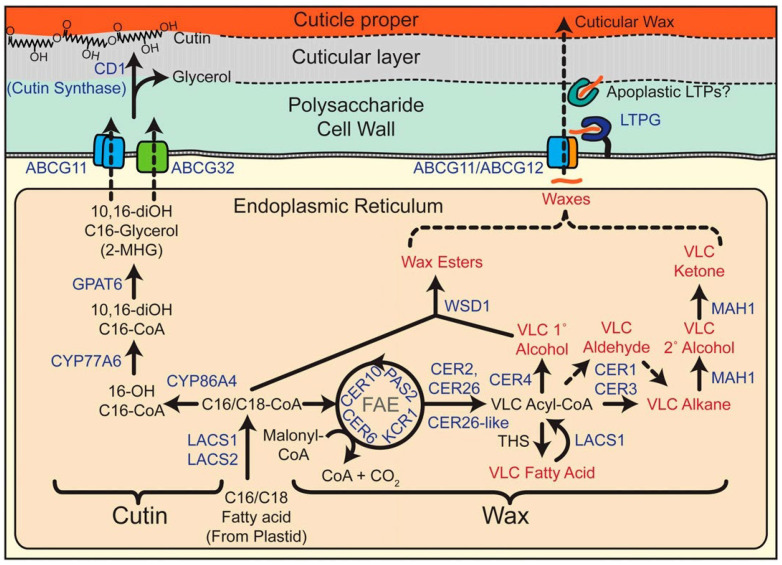
Cutin and wax biosynthetic pathways [34].

Fatty acids serve as the precursors for both cutin and wax biosynthesis. Specifically, C16:0 and C18:x hydroxy fatty acids act as the main precursors for cutin synthesis, whereas C16 and C18 fatty acids are utilized for wax production. During synthesis, these precursors are first generated through the action of various enzymes and are subsequently transported extracellularly, where they undergo polymerization and are deposited into the cuticular membrane [34] (Figure 6).

#### 3.1.1. Biosynthesis and Transport of Cutin

Cutin synthesis involves the formation of C16 and C18 fatty acids, their modification into C16:0 and C18:x hydroxy fatty acids, and the subsequent assembly of cutin monomers [58,59,60,61]. These monomers are then transported extracellularly and polymerized into the cuticular membrane.

(1) Under the catalysis of fatty acid synthase (FAS), acetyl-CoA and malonyl-acyl carrier protein (malonyl-ACP) undergo repeated condensation and elongation to form C16 or C18 fatty acyl-ACPs. These are subsequently hydrolyzed by fatty acyl-ACP thioesterase (FAT) to yield free C16 or C18 fatty acids [62].
(2) Fatty acids are initially activated by long-chain acyl-CoA synthetases (LACSs) to form fatty acyl-CoAs. These activated intermediates can then be oxidized by cytochrome P450 monooxygenases, specifically CYP86A and CYP77A. CYP86A catalyzes hydroxylation at the terminal carbon, whereas CYP77A mediates mid-chain hydroxylation [63].
(3) Glycerol-3-phosphate acyltransferases (GPATs) catalyze the transfer of an acyl group from acyl-CoA to glycerol-3-phosphate, yielding monoacylglycerol. The typical end product of cutin biosynthesis is 2-monoacylglycerol; however, in the presence of 10,16-dihydroxyhexadecanoic acid, the end product is 2-hydroxy-hexadecanoic monoacylglycerol (2-MHG) [64]. Among cutin monomers in pepper fruit, 10,16-dihydroxyhexadecanoic acid is the most abundant and is likely a fundamental unit for the assembly of cutin polyesters [40].

#### 3.1.2. Biosynthesis and Transport of Wax

**Figure 7:**
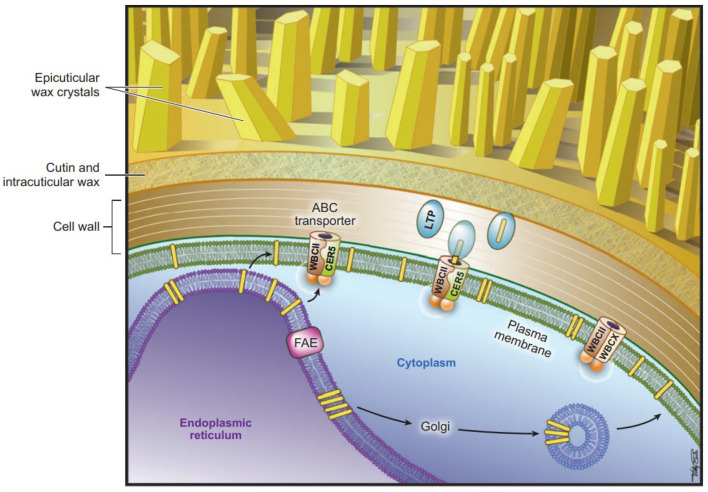
The model of cuticle wax export from epidermal cell to the cuticle [56].

During wax synthesis (Figure 7) [56], C16–C18 fatty acids are initially released from acyl-ACPs and exported to the cytoplasm as free fatty acids. Long-chain acyl-CoA synthetases (LACS) located on the outer plastid membrane subsequently convert these fatty acids into C16 or C18 acyl-CoAs [65]. Further processing occurs in the endoplasmic reticulum (ER), where *LACS1* and *LACS2* participate in the biosynthesis of waxes and cutin. Within the ER membrane, the synthesized C16 and C18-CoAs undergo elongation to VLCFAs under the catalysis of *LACS1* and *LACS2*. Subsequently, ER-associated fatty acid elongases (FAEs) sequentially add C2 units to malonyl-CoA, generating VLCFAs with carbon chain lengths of C20-C34 [66].

VLCFAs are modified into various wax components via two major biosynthetic pathways [67]. The acyl-reduction pathway generates primary alcohols and lipids with even carbon numbers, whereas the decarbonylation pathway yields fatty aldehydes, alkanes, secondary alcohols, and ketones. Studies on Arabidopsis stems indicate that nearly 90% of wax is synthesized through the decarbonylation pathway [68].

In epidermal cells, cuticular waxes are synthesized on the ER and must be transported to the plasma membrane, traverse it, and subsequently cross the cell wall to be assembled into the cuticle [56]. The export from the plasma membrane to the apoplast is mediated by ATP-binding cassette (ABC) transporters and lipid transfer proteins (LTPs).

### 3.2. Regulatory Genes of Wax and Cutin

Research in the classic model plant Arabidopsis thaliana has led to milestone progress in deciphering the genetic regulatory networks of wax and cutin biosynthesis. Numerous reported mutants with altered glossiness, including *cer* [69], *ltp* [70], *wax* [71], *fdh* [72], *lacs2* [73]. These genes govern gloss phenotypes by modulating diverse processes, including the trafficking and secretion of wax components, the inhibition of very-long-chain fatty acid (e.g., C28) elongation, and the transfer of lipid precursors.

Using transcriptome sequencing technology, a study of the pepper cuticle defective mutant “*Pcd1*” identified 3781 differentially expressed genes, of which approximately 1500 DEGs were significantly enriched in the cuticle synthesis pathway [74]. Further studies showed that the recessive gene *CaFCD1* located on chromosome 12 causes base substitutions during the development of pepper fruit, leading to premature termination of transcription, thereby affecting the biosynthesis of cutin and wax in pepper fruit [75].

### 3.3. Carotenoids Biosynthesis and Transport

**Figure 8:**
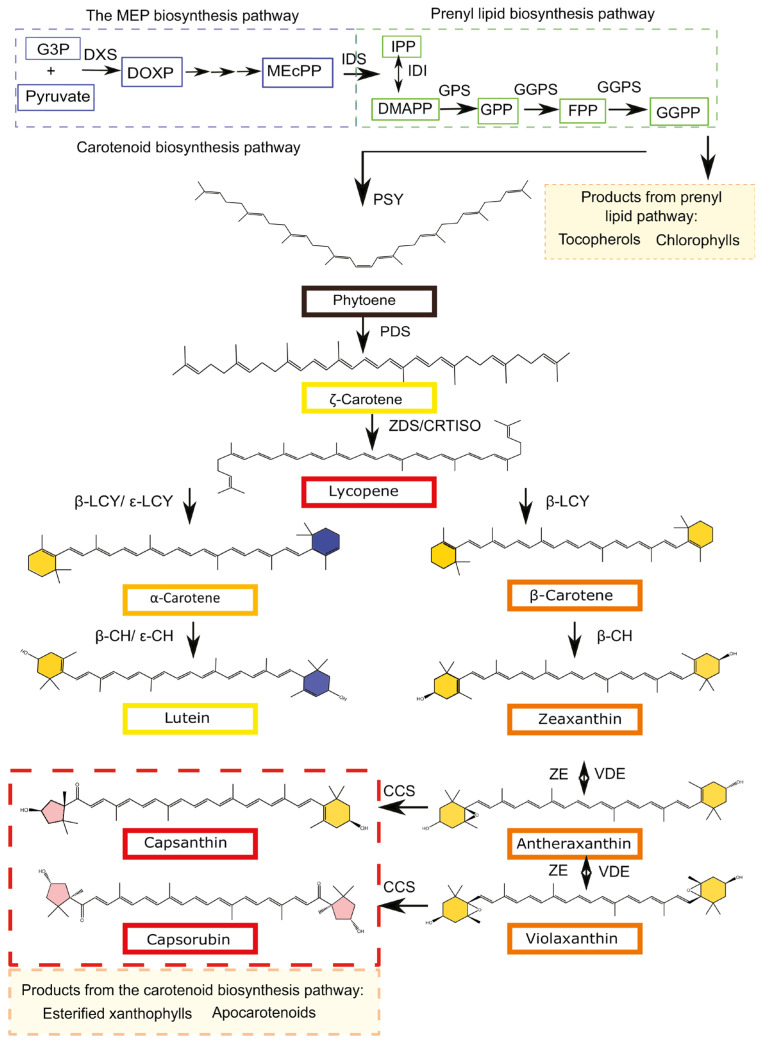
Carotenoid biosynthesis pathway.

The carotenoid biosynthetic pathway in pepper fruit is well-established (Figure 8) [50]. The carotenoid precursor compound isopentenyl diphosphate (IPP) is catalyzed by a series of enzymes to produce geranyl pyrophosphate (GPP). GPP is then condensed by geranylgeranyl pyrophosphate synthase (GGPS) to form geranylgeranyl pyrophosphate (GGPP) [76]. GGPP is a direct precursor for carotenoid biosynthesis, catalyzed by phytoene synthase (PSY) to form phytoene. Phytoene is the first carotenoid in the carotenoid biosynthesis pathway. Phytoene desaturase (PDS) catalyzes the formation of phytofluene, ζ-carotene, and finally ζ-carotene desaturase (ZDS) to produce lycopene.

Lycopene serves as a branch point for two key pathways. One branch leads to α-carotene through the sequential catalysis of lycopene-β-cyclase (LCYb) and lycopene-ε-cyclase (LCYe). The other branch yields β-carotene via the action of LCYb [77]. Subsequently, α-carotene is converted to lutein (ε branch) by carotene ε-hydroxylase (CYP97C) and β-carotene hydroxylase (CHYB).

β-Carotene is hydroxylated by CHYB to yield β-cryptoxanthin and zeaxanthin. Zeaxanthin is subsequently converted to violaxanthin via two sequential epoxidation reactions catalyzed by zeaxanthin epoxidase (ZEP), with antheraxanthin as an intermediate. Violaxanthin is then transformed into neoxanthin by neoxanthin synthase, completing the β branch. The interconversions between zeaxanthin, antheraxanthin, and violaxanthin are reversible and are mediated by violaxanthin de-epoxidase [78]. In red pepper fruit, capsanthin synthase (CCS) catalyzes the conversion of antheraxanthin to capsanthin and violaxanthin to capsorubin [79].

### 3.4. Regulatory Genes of Carotenoids

Current studies on carotenoid regulation in pepper fruit primarily focus on the transcriptional regulation and protein-level modulation of carotenoid biosynthetic genes across the metabolic pathway. Key enzymatic components under investigation include *GGPS*, *PSY*, *PDS*, *ZDS*, *LCYb*, *LCYe*, *Crtz-2*, *CCS*, etc [80,81]. These studies aim to elucidate the regulatory genetics underlying chromatic transitions during fruit maturation. Key genes, such as *PSY*, *LCYB*, and *CCS*, have been cloned and functionally validated by gene silencing in pepper color mutants [82]. Allelic variation, particularly loss-of-function mutations, produces distinct characteristics of carotenoid accumulation (Table 2) [83]. The regulatory roles of specific transcription factors in the carotenoid biosynthetic pathway have been characterized, including *MYB306* [84], *DIVARICATA1* [85], *BBX20* [86]. In addition, *CaERF82*, *CaERF97*, *CaERF66*, *CaERF107a* and *CaERF101* have been functionally linked to the regulation of carotenoid metabolism [85].

The color of mature pepper fruit is governed by three independent loci, *C1*, *C2*, and *Y* [87], which encode *PRR2*, *PSY1*, and *CCS* genes, respectively. Red (*Y*) is dominant to yellow or orange (*y*), and its expression level governs red color intensity. 1-deoxy-d-xylulose-5-phosphate synthase (DXS), a rate-limiting enzyme in the methylerythritol phosphate (MEP) pathway, likewise modulates the intensity of fruit red [50]. At the orange and red stages of pepper development, Wang et al. [88] observed a significant increase in carotenoid accumulation, accompanied by the upregulation of genes involved in carotenoid biosynthesis, such as *CaPSY*, *CaPDS*, *CaZISO*, *CaZDS*, *CaLCYB*, *CaCHYB*, *CaZEP*, and *CaCCS*.

A functional *C1* allele confers enhanced chromatic intensity and surface gloss to pepper fruits compared to loss-of-function allele [89]. The chromatic parameter L* correlated exclusively with the expression of *LYC-B* and *GGPS*. In contrast, the a* value exhibited stronger associations with the expression of key carotenogenic genes, most notably *CCS*, than either L* or b* [90].

## 4. Pepper Fruit Glossiness After Harvest

Pepper fruit, being non-climacteric vegetables, undergo complex physiological and biochemical deterioration post-harvest. This process primarily involves oxidative reactions between reactive oxygen species (ROS) and cellular membranes, resulting in structural and functional membrane damage. Macroscopically, these changes manifest as fruit softening, pericarp pitting, surface discoloration, and eventual decay [91,92].

Lipoxygenases (LOXs) are pivotal enzymes in plant lipid metabolism that modulate fruit color transition and softening. Studies demonstrate that LOX-mediated peroxidative degradation of membrane lipids compromises cellular compartmentalization, leading to membrane integrity loss and subsequent release of phenolic compounds. This cascade consequently drives quality deterioration in horticultural crops, manifesting as senescence, chromatic alterations, and moisture deficit [93]. During early post-harvest deterioration of pepper fruit, increased LOXs activity oxidatively degrades carotenoids, resulting in approximately 30% loss within 24 h and significant chromatic degradation [94]. Concurrently, elevated LOXs levels accelerate free linoleic acid metabolism, compromising membrane integrity and driving rapid water loss [95].

During extended storage, green pepper fruits exhibited progressive pericarp darkening and significantly increased chromatic indices. By day 12, specimens displayed obvious water loss and shrinkage [96]. Holden et al. analyzed 13 pepper cultivars and revealed a correlation between carotenoid retention capacity and fruit surface morphology: genotypes with low retention showed pericarp wrinkling and cracking upon desiccation, whereas high-retention genotypes maintained smooth surfaces [97]. Furthermore, enhanced carotenoid retention correlated with elevated cutin monomer content and increased exocarp thickness, suggesting the cuticle preserves postharvest pigmentation by protecting carotenoids from degradation.

## 5. Conclusions and Perspectives

Pepper fruit glossiness constitutes a multifactorial phenotypic trait emerging from the structure of cutin, epicuticular wax optical properties, and pigment deposition density. Crystalline ordering of long-chain alkanes (e.g., C31) enhances specular highlight anisotropy while reducing diffuse scattering contrast, thereby creating a high-gloss surface. Meanwhile, an imbalance in wax composition or disordered crystalline arrangements amplify light scattering, resulting in a matte phenotype. Throughout fruit maturation, changes in the ratio of wax components to cutin monomers directly influence the fruit’s refractive index. Carotenoid concentration governs chromatic coordinates (L*, a*, b*), serving as the primary factor of pepper fruit hue and color value. This review synthesizes regulatory mechanisms underlying fruit glossiness, focusing on cuticular structure, wax biosynthesis pathways, and carotenoid metabolism with their associated genetic determinants.

Future research on pepper fruit glossiness should focus on: (1) Based on precision phenotyping, using GWAS to identify significant SNP loci and candidate genes controlling pepper pericarp glossiness, and systematically elucidating the genetic mechanisms underlying pepper fruit glossiness; (2) Developing glossiness models based on machine vision, establishing multimodal data fusion models, and achieving correlative prediction between glossiness phenotypes and genotypes. These advances will establish a mechanistic framework for molecular breeding of high-gloss pepper varieties, accelerating development of cultivars with enhanced surface optics and physiological performance for improved commercial value.

## Figures and Tables

**Table 1:** Comparative Summary of Glossiness Measurement Methods.

Methods	Advantages	Disadvantages	Applicable Scenarios
Gloss Meter	High standardization level, easy operation, non-destructive testing	Only reflects macroscopic gloss, fruit shape limitation	Breeding screening, market grading
Optical Imaging and Machine Learning	High-throughput detection, strong real-time performance	Dependence on data annotation, environmental sensitivity	Industrial automation
Optical Coherence Tomography	non-invasive detection, 3D imaging	resolution limitation, high equipment cost	Storage and preservation research, variety improvement,
Colorimeter	Compact size for field operation, cost-effective	Low spectral resolution, relies on manual positioning of measurement areas	screening of color diversity in pepper germplasm resources; rapid on-site detection during fresh pepper procurement
Computer Vision System	Non-contact high-throughput analysis, multi-parameter fusion	High equipment cost, requires professional algorithm development and large amounts of training data	Pepper phenomics research, real-time detection in intelligent sorting production lines

**Table 2:** Genetic and molecular findings for key carotenoid and chlorophyll biosynthesis pathway genes [83].

Locus	Chr	Protein	Trait/Function
*PSY1/c2*	4	Phytoene synthase 1	Orange fruit color
*CCS/y*	6	Capsanthin capsorubin synthase	Yellow fruit color
*PRR2/c1*	1	Pseudo-response regulator 2	Lighter fruit color (red, yellow, orange)
*PSY2*	2	Phytoene synthase 2	Yellow fruit color
*LCYB*	5	Phytoene synthase 2	Fruit color variation (pink to orange)
*CrtZ-2/* *BCH/* *CHY2*	3	β-Carotene hydroxylase 2	Orange fruit color
*ZEP/Or*	2	β-Carotene hydroxylase 2	Orange/yellow mature fruit color
*BBX20*	6	B-box (BBX) C-zinc-finger transcription factor (TF)	Regulates a carotenoid synthesis pathway gene (*CCS*); *BBX20* silencing results in orange fruits
*PP2C35*	10	Type 2 C protein phosphatases	Green stripes on fruit surface
*LOL1* *(pc1)*	1	LOL1 (LSD ONE LIKE1) zinc-finger TF	Null mutation in *LOL1* determines light-green fruit color, chloroplast size, and chlorophyll content
*SGR1 (c)*	1	Stay-green (SGR) gene, encodes a Magnesium dechelatase	Responsible for the stay-green phenotype
*GLK2* *(pc10)*	10	GOLDEN2-LIKE TF	Determines light- and dark-green fruit color, chloroplast size, and chlorophyll content

## Data Availability

The original data presented in the study are openly available in TabShare at https://doi.org/10.1016/j.cpb.2023.100303, [83].

## Abbreviations

The following abbreviations are used in this manuscript:

VLCFAs	very-long-chain fatty acids
ROS	reactive oxygen species
LOXs	lipoxygenases
DXS	1-deoxy-d-xylulose-5-phosphate synthase
MEP	methylerythritol phosphate
IPP	isopentenyl diphosphate
GPP	geranyl pyrophosphate
GGPS	geranylgeranyl pyrophosphate synthase
GGPP	geranylgeranyl pyrophosphate
PSY	phytoene synthase
PDS	Phytoene desaturase
ZDS	ζ-carotene desaturase

## References

[1] Zou X.X., Ma Y.Q., Dai X.Z., Li X.F., Yang S. (2020). Spread and Industry Development of Pepper in China. Acta Hortic. Sin..

[2] Barboza G.E., García C.C., de Bem Bianchetti L., Romero M.V., Scaldaferro M. (2022). Monograph of wild and cultivated chili peppers (*Capsicum* L., *Solanaceae*). PhytoKeys..

[3] Cheng J.W., Lai Z.P., Dong J.C., Tan C., Wu Z.M., Liao Y., Chen C.M., Song Z., Chen M.X., Cui J.J. (2024). Research Progress in Genomics of Pepper (*Capsicum* spp.). Guangdong Agric. Sci..

[4] Blanke M. (2024). Loss of Gloss: A Fresh Look at Freshness. Recent Advances in Postharvest Technologies.

[5] Mangal M., Srivastava A., Tomar B.S. (2018). Genetic and molecular regulation of colour and pungency in Hot pepper (*Capsicum* spp.): A review. Indian J. Agric. Sci..

[6] Liang B., Sun Y., Wang J., Zheng Y., Zhang W., Xu Y., Li Q., Leng P. (2021). Tomato protein phosphatase 2C influences the onset of fruit ripening and fruit glossiness. J. Exp. Bot..

[7] Zhang M., Zhang P., Lu S., Ou-Yang Q., Zhu-Ge Y., Tian R., Jia H., Fang J. (2021). Comparative analysis of cuticular wax in various grape cultivars during berry development and after storage. Front. Nutr..

[8] Gao L., Cao J., Gong S., Hao N., Du Y., Wang C., Wu T. (2023). The COPII subunit *CsSEC23* mediates fruit glossiness in cucumber. Plant J..

[9] Liu X., Ge X., An J., Liu X., Ren H. (2023). *CsCER6* and *CsCER7* influence fruit glossiness by regulating fruit cuticular wax accumulation in cucumber. Int. J. Mol. Sci..

[10] Yang H., Mei W., Wan H., Xu R., Cheng Y. (2021). Comprehensive analysis of *KCS* gene family in Citrinae reveals the involvement of *CsKCS2* and *CsKCS11* in fruit cuticular wax synthesis at ripening. Plant Sci..

[11] Wang H., Nie Z., Wang T., Yang S., Zheng J. (2024). Comparative transcriptome analysis of eggplant (*Solanum melongena* L.) peels with different glossiness. Agronomy.

[12] Yang X., Zhang W., Li Y., He H., Bie B., Ren G., Zhao J., Wang Y., Nie J., Pan J. (2014). High-resolution mapping of the dull fruit skin gene D in cucumber (*Cucumis sativus* L.). Mol. Breed..

[13] Zhai X., Wu H., Wang Y., Zhang Z., Shan L., Zhao X., Wang R., Liu C., Weng Y., Wang Y. (2022). The fruit glossiness locus, *dull fruit* (*D*), encodes a C_2_H_2_-type zinc finger transcription factor, CsDULL, in cucumber (*Cucumis sativus* L.). Hortic. Res..

[14] Hao Y., Luo H., Wang Z., Lu C., Ye X., Wang H., Miao L. (2024). Research progress on the mechanisms of fruit glossiness in cucumber. Gene.

[15] Dong S., Li J., Zhang J., Song L., Wang Y., Zhao L., Chen J., Brotman Y., Zhao T. (2025). Exploring the sheen: A review of research advances on fruit glossiness. Front. Plant Sci..

[16] Honson V., Huynh-Thu Q., Arnison M., Monaghan D., Isherwood Z.J., Kim J. (2020). Effects of shape, roughness and gloss on the perceived reflectance of colored surfaces. Front. Psychol..

[17] (2025). Determination of the Specular Glossiness of Cowhorn/Sheephorn Pepper—Glossmeter Test Method. https://www.ttbz.org.cn/Home/Standard?searchType=3&key=%E5%85%89%E6%B3%BD%E5%BA%A6.

[18] Zhou R., Zhao T.M., Zhao L.P., Wang Y.L., Song L.X., Yu W.G. (2018). High-glossiness tomato breeding and fruit surface glossiness. Jiangsu J. Agric. Sci..

[19] Mendoza F., Dejmek P., Aguilera J.M. (2010). Gloss measurements of raw agricultural products using image analysis. Food Res. Int..

[20] Althaus B., Blanke M. (2020). Non-destructive, opto-electronic determination of the freshness and shrivel of Bell pepper fruits. J. Imaging.

[21] Kasampalis D.S., Tsouvaltzis P., Ntouros K., Gertsis A., Gitas I., Siomos A.S. (2021). The use of digital imaging, chlorophyll fluorescence and Vis/NIR spectroscopy in assessing the ripening stage and freshness status of bell pepper fruit. Comput. Electron. Agric..

[22] Wang L.Q., Dai X.Z. (2009). Application of colorimeter for testing its color change during the development of hot pepper (*Capsicum annuum* L.) fruit. J. China Capsicum.

[23] Li L., Ding W.K. (2017). Chili recognition based on convolution neural network. J. Tianjin Univ. Technol..

[24] Trivedi P., Nguyen N., Klavins L., Kviesis J., Heinonen E., Remes J., Jokipii-Lukkari S., Klavins M., Karppinen K., Jaakola L. (2021). Analysis of composition, morphology, and biosynthesis of cuticular wax in wild type bilberry (*Vaccinium myrtillus* L.) and its glossy mutant. Food Chem..

[25] Wang X. (2023). Development of Hairy Gene Markers and Screening of Tomato Elite Inbred Lines. Master’s Thesis.

[26] Hao N., Yao H., Suzuki M., Li B., Wang C., Cao J., Fujiwara T., Wu T., Kamiya T. (2024). Novel lignin-based extracellular barrier in glandular trichome. Nat. Plants.

[27] Gebretsadik K., Qiu X., Dong S., Miao H., Bo K. (2021). Molecular research progress and improvement approach of fruit quality traits in cucumber. Theor. Appl. Genet..

[28] Nakamura S., Inoue S., Igarashi Y., Sato H., Mizokami Y. (2024). Analysis of Gloss Unevenness and Bidirectional Reflectance Distribution Function in Specular Reflection. J. Imaging.

[29] Shen Y., Mao L., Zhou Y., Sun Y., Lv J., Deng M., Liu Z., Yang B. (2024). Transcriptome analysis reveals key genes involved in trichome formation in pepper (*Capsicum annuum* L.). Plants.

[30] Liu X.D., Yan J.R., Wang P.Y., Zhang J.Y., Shen H.Y. (2015). Genetic Inheritance and Gene Mapping of Pepper Trichomes Traits. China Veg..

[31] Ma D.H., Pang J.A., Wen X.G., Li S.J., Huo Z.R., Lin S.Q. (2002). Study on Characteristic of Glabrous Cucumber (*Cucumis sativus* L.). Acta Hortic. Sin..

[32] Kim H.J., Han J.H., Kwon J.K., Park M., Kim B.D., Choi D. (2010). Fine mapping of pepper trichome locus 1 controlling trichome formation in *Capsicum annuum* L. CM334. Theor. Appl. Genet..

[33] Jeffree C.E. (2006). The fine structure of the plant cuticle. Biol. Plant Cuticle.

[34] Yeats T.H., Rose J.K.C. (2013). The formation and function of plant cuticles. Plant Physiol..

[35] Bourgault R., Matschi S., Vasquez M., Qiao P., Sonntag A., Charlebois C., Mohammadi M., Scanlon M.J., Smith L.G., Molina I. (2020). Constructing functional cuticles: Analysis of relationships between cuticle lipid composition, ultrastructure and water barrier function in developing adult maize leaves. Ann. Bot..

[36] Petit J., Bres C., Mauxion J.P., Bakan B., Rothan C. (2017). Breeding for cuticle-associated traits in crop species: Traits, targets, and strategies. J. Exp. Bot..

[37] Kolattukudy P.E. (1980). Biopolyester membranes of plants: Cutin and suberin. Science.

[38] Barthlott W., Neinhuis C., Cutler D., Ditsch F., Meusel I., Theisen I., Wilhelmi H. (1998). Classification and terminology of plant epicuticular waxes. Bot. J. Linn. Soc..

[39] Koch K., Ensikat H.J. (2008). The hydrophobic coatings of plant surfaces: Epicuticular wax crystals and their morphologies, crystallinity and molecular self-assembly. Micron.

[40] Ge S., Qin K., Ding S., Yang J., Jiang L., Qin Y., Wang R. (2022). Gas chromatography–mass spectrometry metabolite analysis combined with transcriptomic and proteomic provide new insights into revealing cuticle formation during pepper development. J. Agric. Food Chem..

[41] De Rijke E., Fellner C., Westerveld J., Lopatka M., Cerli C., Kalbitz K., De Koster C.G. (2015). Determination of n-alkanes in *C. annuum* (bell pepper) fruit and seed using GC-MS: Comparison of extraction methods and application to samples of different geographical origin. Anal. Bioanal. Chem..

[42] Ikedou K., Yamamoto H., Nagashima H., Nemoto N., Tashiro K. (2005). Crystal structures of n-alkanes with branches of different size in the middle. J. Phys. Chem. B.

[43] Cai Y., Kiyokawa H., Nagai T., Haghzare L., Arnison M., Kim J. (2023). Effects of specular roughness on the perception of color and opacity. J. Opt. Soc. Am. A.

[44] Natarajan P., Akinmoju T.A., Nimmakayala P., Lopez-Ortiz C., Garcia-Lozano M., Thompson B.J., Stommel J., Reddy U.K. (2020). Integrated metabolomic and transcriptomic analysis to characterize cutin biosynthesis between low-and high-cutin genotypes of *Capsicum chinense* Jacq. Int. J. Mol. Sci..

[45] Liu Y., Lv J., Liu Z., Wang J., Yang B., Chen W., Qu L., Dai X., Zhang Z., Zou X. (2020). Integrative analysis of metabolome and transcriptome reveals the mechanism of color formation in pepper fruit (*Capsicum annuum* L.). Food Chem..

[46] D’Souza M.C., Singha S., Ingle M. (1992). Lycopene concentration of tomato fruit can be estimated from chromaticity values. HortScience.

[47] Zhang Z.H., Cao Y.C., Yu H.L., Wang L.H., Zhang B.X. (2019). Genetic control and metabolite composition of fruit quality in Capsicum. Acta Hortic. Sin..

[48] Li L.Y., Wu L.L., Huang H.R., Bozhi Y.A.N.G., Shudong Z.H.O.U. (2024). Comparative Color and Quality Analyses of Color Mutants and Wild-Type of Pepper at Different Developmental Stages. Food Sci..

[49] Guo Y.M., Duan X.D., Bai J.J., Wang J.E. (2021). Extraction of anthocyanin from Ormamental Pepper fruits and correlation analysis of fruit color. J. Shanxi Agric. Univ. Nat. Sci. Ed..

[50] Berry H.M., Rickett D.V., Baxter C.J., Enfissi E.M., Fraser P.D. (2019). Carotenoid biosynthesis and sequestration in red chilli pepper fruit and its impact on colour intensity traits. J. Exp. Bot..

[51] He J.Y., An Y., Li J.X., Xian J.Q., Zhou D.F., Wang J.E. (2025). Fruit Color Phenotype and Pigment Accumulation Characteristics of Chili Peppers (*Capsicum annuum* L.). Acta Agric. Boreali-Occident. Sin..

[52] Tao X.L., Zhu H.X., Zhang Y.X., Liu M.X. (2024). Quality Characteristics and Correlation Analysis of Processed Peppers Based on Principal Component Analysis and Cluster Analysis. Food Res. Dev..

[53] Gong X.F., Chen X., Li H., Xu Y., Song Z.F. (2022). Non-destructive quality detection method and canonical correlation analysis of processed pepper. Jiangsu Agric. Sci..

[54] Dong S.C., Zhang J.W., Ling J.Y., Hong J., Xie Z.X., Zhang S.J., Song L.X., Wang Y.L., Zhao T.M., Zhao L.P. (2024). Evaluation of fruit glossiness in tomato population and genome-wide association analysis. Jiangsu Agric. Sci..

[55] Jetter R., Kunst L., Samuels A.L. (2006). Composition of plant cuticular waxes. Annual Plant Reviews Volume 23: Biology of the Plant Cuticle.

[56] Samuels L., DeBono A., Lam P., Wen M., Jetter R., Kunst L. (2008). Use of Arabidopsis eceriferum mutants to explore plant cuticle biosynthesis. J. Vis. Exp. JoVE.

[57] Beisson F., Koo A.J., Ruuska S., Schwender J., Pollard M., Thelen J.J., Paddock T., Salas J.J., Savage L., Milcamps A. (2003). Arabidopsis genes involved in acyl lipid metabolism. A 2003 census of the candidates, a study of the distribution of expressed sequence tags in organs, and a web-based database. Plant Physiol..

[58] Li-Beisson Y., Shorrosh B., Beisson F., Andersson M.X., Arondel V., Bates P.D., Baud S., Bird D., DeBono A., Durrett T. (2013). Acyl-lipid metabolism. Arab. Book/Am. Soc. Plant Biol..

[59] Samuels L., Jetter R., Kunst L. (2005). First steps in understanding the export of lipids to the plant cuticle. Plant Biosyst.-Int. J. Deal. All Asp. Plant Biol..

[60] Kurdyukov S., Faust A., Nawrath C., Bar S., Voisin D., Efremova N., Franke R., Schreiber L., Saedler H., Meétraux J. (2006). The epidermis-specific extracellular BODYGUARD controls cuticle development and morphogenesis in *Arabidopsis*. Plant Cell.

[61] Yeats T.H., Martin L.B.B., Viart H.M.F., Isaacson T., He Y., Zhao L., Matas A.J., Buda G.J., Domozych D.S., Clausen M.H. (2012). The identification of cutin synthase: Formation of the plant polyester cutin. Nat. Chem. Biol..

[62] García-Coronado H., Tafolla-Arellano J.C., Hernández-Oñate M.Á., Burgara-Estrella A.J., Robles-Parra J.M., Tiznado-Hernández M.E. (2022). Molecular biology, composition and physiological functions of cuticle lipids in fleshy fruits. Plants.

[63] Fich E.A., Segerson N.A., Rose J. (2016). The plant polyester cutin: Biosynthesis, structure, and biological roles. Annu. Rev. Plant Biol..

[64] Duan R.J., Wang A.D., Chen G.X. (2017). Advances in Study of Plant Cuticle Genes. Chin. Bull. Bot..

[65] Borisjuk N., Hrmova M., Lopato S. (2014). Transcriptional regulation of cuticle biosynthesis. Biotechnol. Adv..

[66] Lee S.B., Suh M.C. (2015). Advances in the understanding of cuticular waxes in *Arabidopsis thaliana* and crop species. Plant Cell Rep..

[67] Zhang Y.L., You C.X., Li Y.Y., Hao Y.J. (2020). Advances in biosynthesis, regulation, and function of apple cuticular wax. Front. Plant Sci..

[68] Millar A.A., Clemens S., Zachgo S., Giblin E.M., Taylor D.C., Kunst L. (1999). *CUT1*, an Arabidopsis gene required for cuticular wax biosynthesis and pollen fertility, encodes a very-long-chain fatty acid condensing enzyme. Plant Cell.

[69] Alexander L.E., Gilbertson J.S., Xie B., Song Z., Nikolau B.J. (2021). High spatial resolution imaging of the dynamics of cuticular lipid deposition during Arabidopsis flower development. Plant Direct.

[70] Ni F., Yang M., Chen J., Guo Y., Wan S., Zhao Z., Yang S., Kong L., Chu P., Guan R. (2024). BnUC1 is a key regulator of epidermal wax biosynthesis and lipid transport in *Brassica napus*. Int. J. Mol. Sci..

[71] Chen X., Goodwin S.M., Boroff V.L., Liu X., Jenks M.A. (2003). Cloning and characterization of the *WAX2* gene of Arabidopsis involved in cuticle membrane and wax production. Plant Cell.

[72] Yephremov A., Wisman E., Huijser P., Huijser C., Wellesen K., Saedler H. (1999). Characterization of the *FIDDLEHEAD* gene of Arabidopsis reveals a link between adhesion response and cell differentiation in the epidermis. Plant Cell.

[73] Schnurr J., Shockey J., Browse J. (2004). The acyl-CoA synthetase encoded by *LACS2* is essential for normal cuticle development in Arabidopsis. Plant Cell.

[74] Yi T. (2020). Cuticle Composition Analysis and Transcriptome Study of Pepper Keratin Deficiency Mutant “*Pcd1*”. Master’s Thesis.

[75] Wang J., Shan Q., Yi T., Ma Y., Zhou X., Pan L., Miao W., Zou X., Xiong C., Liu F. (2023). Fine mapping and candidate gene analysis of *CaFCD1* affecting cuticle biosynthesis in *Capsicum annuum* L.. Theor. Appl. Genet..

[76] Guzman I., Hamby S., Romero J., Bosland P.W., O’Connell M.A. (2010). Variability of carotenoid biosynthesis in orange colored *Capsicum* spp.. Plant Sci..

[77] del Rocío Gómez-García M., Ochoa-Alejo N. (2013). Biochemistry and molecular biology of carotenoid biosynthesis in chili peppers (*Capsicum* spp.). Int. J. Mol. Sci..

[78] Filyushin M.A., Dyachenko E.A., Efremov G.I., Kochieva E.Z., Shchennikova A.V. (2021). Variability and expression pattern of phytoene synthase (PSY) paralogs in pepper species. Russ. J. Genet..

[79] Berry H. (2015). Elucidation of the Molecular and Biochemical Mechanisms Associated with Colour Intensity and Colour Retention in Fresh and Dry Chilli Peppers. Ph.D. Thesis.

[80] Xue Q., Zhang Q., Zhang A., Li D., Liu Y., Xu H., Yang Q., Liu F., Han T., Tang X. (2024). Integrated metabolome and transcriptome analysis provides clues to fruit color formation of yellow, orange, and red bell pepper. Sci. Rep..

[81] Tian S.L., Li Z., Li L., Shah S.N.M., Gong Z.H. (2017). Analysis of tandem repeat units of the promoter of capsanthin/capsorubin synthase (*Ccs*) gene in pepper fruit. Physiol. Mol. Biol. Plants.

[82] Tian S.L., Li L., Shah S.N.M., Gong Z.H. (2015). The relationship between red fruit colour formation and key genes of capsanthin biosynthesis pathway in *Capsicum annuum*. Biol. Plant..

[83] Venkatesh J., Lee S.Y., Back S., Kim T.G., Kim G.W., Kim J.M., Kwon J.K., Kang B.C. (2023). Update on the genetic and molecular regulation of the biosynthetic pathways underlying pepper fruit color and pungency. Curr. Plant Biol..

[84] Ma X., Yu Y.N., Jia J.H., Li Q.H., Gong Z.H. (2022). The pepper MYB transcription factor *CaMYB306* accelerates fruit coloration and negatively regulates cold resistance. Sci. Hortic..

[85] Song J., Sun B., Chen C., Ning Z., Zhang S., Cai Y., Zheng X., Cao B., Chen G., Jin D. (2023). An R-R-type MYB transcription factor promotes non-climacteric pepper fruit carotenoid pigment biosynthesis. Plant J..

[86] Ma J., Dai J., Liu X., Lin D. (2023). The transcription factor CaBBX20 regulates capsanthin accumulation in pepper (*Capsicum annuum* L.). Sci. Hortic..

[87] Hurtado-Hernandez H., Smith P.G. (1985). Inheritance of mature fruit color in *Capsicum annuum* L.. J. Hered..

[88] Wang H., Jia L., Li D., Manzoor M.A., Yan C., Ding Q., Wang W., Hong X., Song T., Jiang H. (2025). Transcriptomic and Metabolomic Analysis Reveals the Mechanism of H18 Pepper Color Change. Agriculture.

[89] Jeong H.B., Kang M.Y., Jung A., Han K., Lee J.H., Jo J., Lee H.Y., An J.W., Kim S., Kang B.C. (2019). Single-molecule real-time sequencing reveals diverse allelic variations in carotenoid biosynthetic genes in pepper (*Capsicum* spp.). Plant Biotechnol. J..

[90] Guo Y., Bai J., Duan X., Wang J. (2021). Accumulation characteristics of carotenoids and adaptive fruit color variation in ornamental pepper. Sci. Hortic..

[91] Kang S.H., Yu M.S., Kim J.M., Park S.K., Lee C.H., Lee H.G., Kim S.K. (2018). Biochemical, microbiological, and sensory characteristics of stirred yogurt containing red or green pepper (*Capsicum annuum* cv. Chungyang) juice. Korean J. Food Sci. Anim. Resour..

[92] Gao L.J., Zeng X.F., He X.H., Yin X.M. (2019). Zeng, Z.H. Research progress on post harvest storage physiology and preservation technology of pepper. South China Agric..

[93] Bin W., Wei L., Xiao Y. (2025). Research Progress in Mechanism and Preventive Technologies for the Browning of Fresh-Cut Fruits and Vegetables. Shipin Kexue.

[94] Maalekuu K., Elkind Y., Leikin-Frenkel A., Lurie S., Fallik E. (2006). The relationship between water loss, lipid content, membrane integrity and LOX activity in ripe pepper fruit after storage. Postharvest Biol. Technol..

[95] Jarén-Galán M., Mínguez-Mosquera M.I. (1999). Effect of pepper lipoxygenase activity and its linked reactions on pigments of the pepper fruit. J. Agric. Food Chem..

[96] Lan Z., Lin Y., Huang J., Akutse K.S. (2023). The color matters: Color regulation mechanism of green pepper fruit after harvest. Fruits.

[97] Holden A.C., Cohen H., Berry H.M., Rickett D.V., Aharoni A., Fraser P.D. (2024). Carotenoid retention during post-harvest storage of Capsicum annuum: The role of the fruit surface structure. J. Exp. Bot..

